# Prognostic Implications of Left Atrial Spontaneous Echo Contrast with Catheter Ablation of Nonvalvular Atrial Fibrillation Patients with Left Atrial Dilation

**DOI:** 10.3390/jcdd9090306

**Published:** 2022-09-15

**Authors:** Changjian Lin, Yangyang Bao, Yun Xie, Yue Wei, Qingzhi Luo, Tianyou Ling, Qi Jin, Wenqi Pan, Yucai Xie, Liqun Wu, Ning Zhang

**Affiliations:** Department of Cardiovascular Medicine, Ruijin Hospital, Shanghai Jiao Tong University School of Medicine, Shanghai 200025, China

**Keywords:** atrial fibrillation, left atrial spontaneous echo contrast, pulmonary vein isolation, thrombus

## Abstract

Background: Left atrial spontaneous echo contrast (LASEC) can be detected by transesophageal echocardiography (TEE) before the catheter ablation of atrial fibrillation (AF), especially in patients with left atrial (LA) dilation. Whether LASEC has prognostic value in predicting the procedure outcomes in patients with an enlarged atrium is unknown. The prognostic implications of LASEC with the catheter ablation of AF patients with LA dilation will be evaluated in this study. Methods: AF patients scheduled to undergo catheter ablation in Ruijin Hospital, Shanghai, China, between January 2018 and June 2020 were screened for this prospective study. All patients underwent TEE before the procedure. Patients with a left atrial diameter (LAD; 45 mm ≤ LAD < 50 mm) and left atrial volume (LAV ≥ 120 mL) were enrolled in this study. The endpoint was AF/atrial tachycardia (AT) recurrence-free survival following a 3-month blanking period after the catheter ablation. All patients were followed up for 18 months. Results: This study included 123 AF patients, who were divided into the LASEC (*n* = 73) and no LASEC (*n* = 50) groups. Baseline patient characteristics were similar in the two groups. At the end of 18 months of follow-up, AF/AT recurrence-free survival was achieved in 33 (45.2%) and 34 (68.0%) patients in the LASEC and no LASEC groups, respectively (*p* = 0.013). In survival analysis, the LASEC group was also associated with a poor outcome of catheter ablation (log-rank test, *p* = 0.011; Cox regression, *p* = 0.015, HR = 2.058, 95%CI = 1.151–3.679). Meanwhile, during the follow-up AF/AT recurrence was observed in 30 (57.7%) and 15 (71.4%) cases in the mild and severe SEC groups, respectively. Ischemic stroke occurred in two patients in the LASEC group. Conclusions: LASEC could be a predictor of the recurrence of AF/AT after catheter ablation in AF patients with LA dilation. The higher the degree of LASEC, the worse the prognosis.

## 1. Introduction

Left atrial spontaneous echo contrast (LASEC) can be detected by transesophageal echocardiography (TEE) before the catheter ablation of atrial fibrillation (AF), especially in patients with left atrial (LA) dilation. LA dilation is a well-established risk factor for predicting outcomes in AF patients after the procedure [1]. Increased LA size is associated with a higher recurrence rate of AF treated with ablation [2]. However, not all AF patients with LA dilation will develop LASEC during TEE. Therefore, the current study investigated whether LASEC may be of prognostic value in AF patients with LA dilation. LASEC is defined as a dynamic smoke-like signal that swirls slowly in a circular pattern within the left atrium and/or left atrial appendage (LA/LAA) and has an optimal gain setting for its distinction from echoes arising from excessive gain [3]. The intensity of LASEC is classified into four grades (0–3) [4]. This study aimed to evaluate the prognostic implications of LASEC with the catheter ablation of nonvalvular AF patients with LA dilation.

## 2. Methods

### 2.1. Study Population

**Figure 1:**
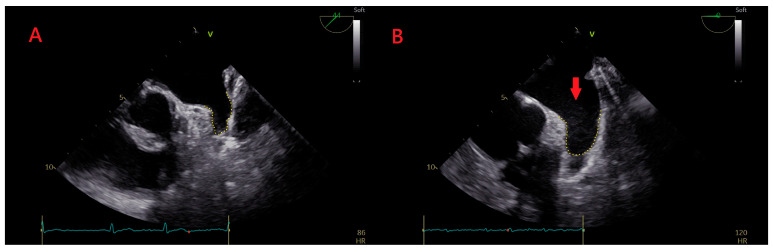
Representative echocardiogram of LASEC. (**A**) No LASEC was detected by TEE. (**B**) LASEC (arrow) was revealed by TEE. V stands for left side, numbers 0 and 44 stand for imaging plane angle.

This prospective study evaluated nonvalvular AF patients who were scheduled to undergo catheter ablation in Ruijin Hospital, Shanghai, China, between January 2018 and June 2020. The presence of LASEC was confirmed by TEE (Figure 1). All patients met the following inclusion criteria: (1) nonvalvular AF or flutter; and (2) left atrial diameter (LAD; 45 mm ≤ LAD < 50 mm) and left atrial volume (LAV; ≥120 mL). The exclusion criteria were as follows: (1) <18 or >80 years old; (2) history of left atrial surgery; (3) TEE-witnessed left atrial thrombus (LAT); (4) uncontrolled hyperthyroidism; (5) pregnancy; and (6) history of myocardial infarction, percutaneous coronary intervention, heart surgery, transient ischemic attack (TIA), or stroke within 3 months before the procedure. All patients provided written informed consent. This study was approved by the Institutional Review Board of Ruijin Hospital, Shanghai Jiao Tong University School of Medicine, and was conducted according to the principles of the Declaration of Helsinki.

### 2.2. Clinical Characteristics

Blood samples from all nonvalvular AF patients were collected and analyzed after admission and before the TEE examination. Baseline demographic characteristics (age and sex), medical history (hypertension, diabetes mellitus, coronary heart disease, AF types, and prior stroke or TIA), laboratory blood biomarkers (creatinine, D-dimer, troponin I, and brain natriuretic peptide), and echocardiographic parameters were recorded. The risk of stroke was assessed using the CHA_2_DS_2_-VASc score, calculated by adding the risk factors of congestive heart failure, hypertension, 65–74 or ≥75 years, diabetes mellitus, stroke or TIA, vascular disease, and female sex. Each parameter was weighted by 1, except for stroke or TIA and age ≥75 years, which were weighted by 2 [5,6].

### 2.3. TEE

TEE was performed to exclude LAT before catheter ablation for AF [7]. TEE was performed under local anesthesia, with continuous monitoring of blood pressure and oximetry. A 5 to 7 MHz multiplane probe was used, and images were independently assessed by two experienced echocardiographers. Images were obtained in multiple standard tomographic planes. The intensity of SEC was classified into four grades from 0 to 3, using the system described by Watanabe et al. [4]. Grade 0 indicated no SEC; grade 1 indicated minimal echogenicity in the LAA; grade 2 indicated moderate echogenicity with a dense swirling pattern in the main cavity; and grade 3 indicated intense echodensity with very slow swirling patterns in the main cavity. A thrombus was defined as a well-circumscribed, highly reflective mass with a texture different from that of the atrial wall and with uniform consistency [8].

### 2.4. Preparation for the Procedure

Antiarrhythmic medications were discontinued before the ablation procedure for at least five half-lives. All patients received standard anticoagulation therapy for at least 1 month before the procedure. The international normalized ratio was maintained at 2–2.5 for patients taking warfarin. Each patient received an electrophysiological study in a conscious state after TEE confirmed the absence of atrial thrombus.

### 2.5. Ablation Procedure

The pulmonary vein was initially isolated by cryoballoon ablation (CBA) or radiofrequency ablation (RFA).

A 28 mm cryoballoon (Arctic Front Advance; Medtronic Inc., Minneapolis, MN, USA) and a circular mapping catheter (Achieve; Medtronic Inc.) were placed following a transseptal puncture for CBA. A three-dimensional (3D) mapping system (EnSite™ Abbott, Saint Paul, MN, USA) was used to perform 3D LA electroanatomic mapping. The circular catheter was used for mapping and recording before and after the pulmonary vein isolation. The cryoballoon was inserted using a transseptal puncture and an over-the-wire delivery technique. Each pulmonary vein received two CB applications, each lasting 3 min. Adjustment of the application time and the necessity of additional freeze were left to the operator’s discretion. During the ablation of the right superior and inferior pulmonary veins, the phrenic nerve function was continuously monitored by pacing to reduce the risk of phrenic nerve paralysis [9].

A detailed description of the catheter ablation protocol has been published previously for RFA [10]. The ablation catheter comprises pulmonary vein isolation consisting of a point-by-point radiofrequency application using a 3D mapping system (CARTO 3; Biosense Webster, Diamond Bar, CA, USA), two long sheaths, one multielectrode mapping catheter, and a 3.5 mm open-irrigated tip catheter (ThermoCool SF or ThermoCool STSF; Biosense Webster).

Once PV isolation was achieved, electrical cardioversion was attempted. For patients whose rhythm could not be converted to the sinus rhythm, the LA roof line lesion was created by the RF catheter or cryoballoon to ease any subsequent electrical cardioversion. The completion of a bidirectional conduction block along the LA roof line was verified using standard electrophysiological methods. Substrate modification such as ablation of complex fractionated atrial electrograms was not performed in this study. Anticoagulation with nonvitamin K antagonist oral anticoagulant (NOAC) or warfarin was continued after ablation for a minimum of 3 months. After 3 months, anticoagulation treatment was administered at the discretion of the treating clinician.

### 2.6. Follow-Up

All patients remained in the hospital for observation for at least one night. The outpatient clinic visits were scheduled at 1, 3, and 6 months, and every 6 months thereafter. A 24-hour Holter monitoring system was the main method for recurrence assessment, which was performed at 1, 3, 6, 12, and 18 months during follow-up. Patients with suspected AF recurrence symptoms were advised to visit the nearest hospital for an electrocardiogram. The endpoint of this study was AF recurrence-free survival during follow-up. AF recurrence was defined as any of the following events: (1) sustained AF (lasting >30 s); (2) atrial flutter or atrial tachycardia (AFL/AT; lasting >30 s); (3) prescription of antiarrhythmic drugs (class I/III); and (4) repeat ablation. Any arrhythmia that occurred during a standard 3-month blanking period (a standard time allowed for arrhythmias related to ablation-induced inflammation to subside) was not considered an AF recurrence. The presence or absence of stroke or TIA, other systemic thromboembolism, major bleeding events, and minor bleeding events were also assessed during the follow-up.

### 2.7. Statistical Analysis

Continuous and categorical variables were expressed as mean ± SD and a percentage, respectively. Continuous variables were compared between two groups using the Student’s t-test or Mann–Whitney U test. Categorical data were analyzed using the chi-square test or Fisher’s exact test, where appropriate. A value of *p* < 0.05 was considered significant for all determinations. All analyses were performed using SAS statistical software version 9.2 (SAS Institute, Cary, NC, USA). Survival analysis with log-rank test and Cox regression was used to identify the significant predictors of arrhythmia recurrence.

## 3. Results

### 3.1. Baseline Characteristics

This study screened 1560 consecutive patients with nonvalvular AF who underwent TEE at Ruijin Hospital, Shanghai, China. Among them, 123 patients were enrolled and assigned to the LASEC (*n* = 73) and no LASEC (*n* = 50) groups. Fifty-two patients in the LASEC group had SEC grade 1 (mild SEC), while twenty-one patients had SEC grades 2 and 3 (severe SEC).

**Figure 2:**
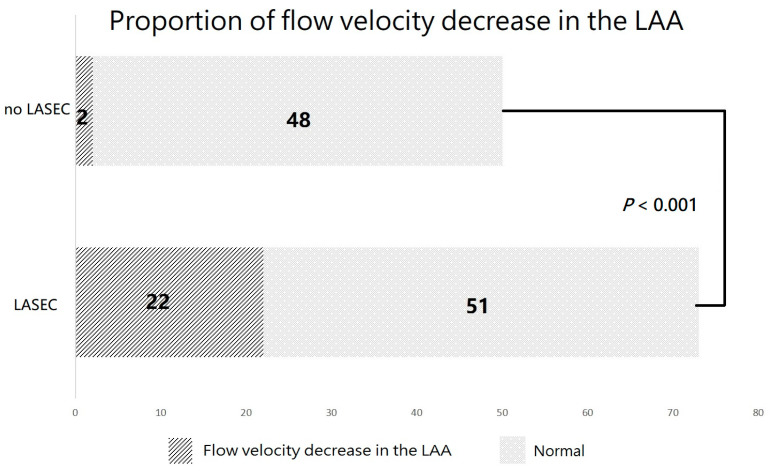
The proportion of the flow velocity decrease in the LAA was higher in the LASEC group than in the no LASEC group (30.1% vs. 4%, *p* < 0.001). LAA, left atrial appendage.

The study population had a mean age of 62.1 ± 9.3 years, and 60.9% were men, with LAD of 47.0 ± 2.1 mm and LAV of 178.4 ± 39.8 cm^3^. The baseline characteristics of the patients in the two groups are shown in Table 1. There were no significant differences in age, sex, body mass index, AF type, or duration of persistent AF between the two groups (*p* > 0.05). Moreover, there were no significant differences in LAV, LAD, left ventricular ejection fraction, serum creatinine, or D-dimer between the two groups (*p* > 0.05). Furthermore, no differences in diabetes mellitus, hypertension, TIA or stroke, coronary heart disease, heart failure, or CHA_2_DS_2_-VASc scores were observed between the two groups (*p* > 0.05). The proportion of the flow velocity decrease in the LAA was higher in the LASEC group than in the no LASEC group (30.1% vs. 4%, *p* < 0.001; Figure 2).

### 3.2. Procedural Details and Medication Use after the Procedure

The mean total procedure time, the left atrial dwell time (the length of time the catheter was present in the left atrium during the procedure), the mean total fluoroscopy time, and the radiation doses were similar between the two groups. The medication use after the procedure was similar in the two groups (Table 2).

### 3.3. Follow-Up

During the blanking period, five and eight patients in the LASEC and no LASEC groups, respectively, underwent electrical cardioversion. No significant difference was observed between the LASEC and no LASEC groups (6.8% vs. 16%, *p* = 0.1).

**Figure 3:**
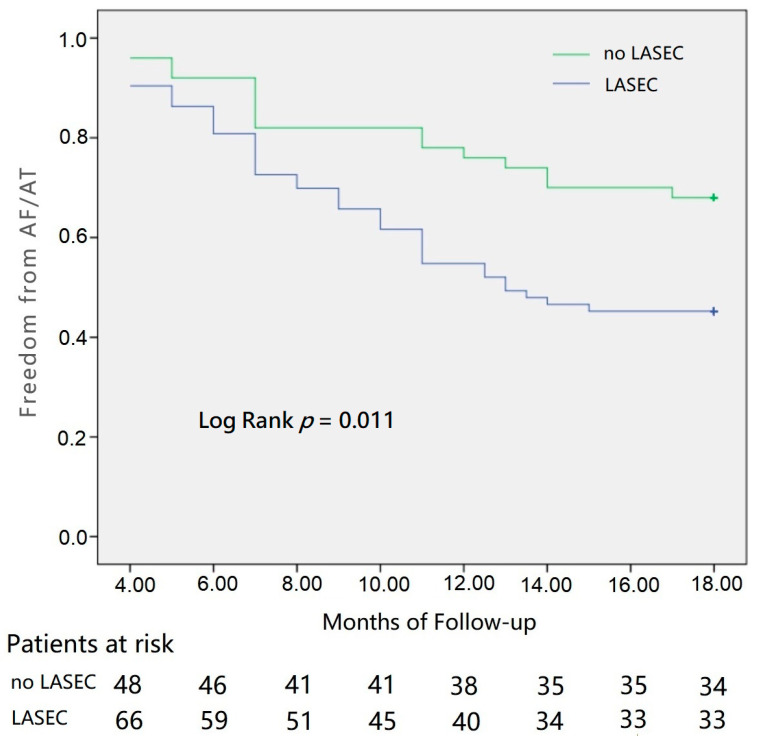
Considering a blanking period of 3 months, AF/AT recurrence in the LASEC and no LASEC groups was 54.8% (40 patients) and 32% (16 patients), respectively, at 18-month follow-up. The difference in LASEC had produced significant variations in AF/AT recurrence-free survival between the two groups.

At 18 months of follow-up, the AF/AT recurrence in the LASEC and no LASEC groups was 54.8% (40 patients) and 32% (16 patients), respectively (Table 3). Kaplan–Meier survival curves reporting each group’s AF/AT recurrence-free survival rates are presented in Figure 3. The difference in LASEC produced significant variations in AF/AT recurrence-free survival between the two groups (log-rank test, *p* = 0.011). The presence of LASEC, long history of AF, increased LAV, and heart failure were associated with poor outcomes in a multivariate Cox regression analysis of AF/AT recurrence-free survival (Table 4).

**Figure 4:**
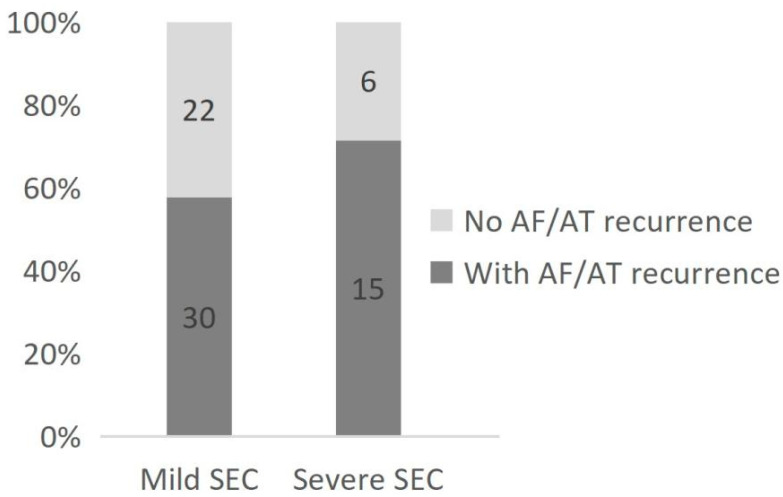
AF/AT recurrence in 30 (57.7%) and 15 (71.4%) cases in the mild and severe SEC groups, respectively.

During the follow-up, AF/AT recurrence was observed in 30 (57.7%) and 15 (71.4%) cases in the mild and severe SEC groups, respectively. Figure 4 reveals that the prognosis declined with increasing SEC degree. Ischemic stroke occurred in two patients in the LASEC group during the follow-up. Severe LASEC was detected on TEE before the ablation procedure for these two patients.

## 4. Discussion

The present study has two main findings. First, catheter ablation outcomes were poor in AF patients with LA dilation and LASEC. LASEC could be a predictor of AF/AT recurrence after a procedure. Second, the prognosis declined with increasing LASEC degree.

TEE is a sensitive method for identifying intracardiac thrombus and LASEC [11]. LASEC can be detected by TEE before the catheter ablation of AF, especially in patients with LA dilation. LA dilation is a well-established risk factor for predicting outcomes in AF patients after the procedure [1]. Increased LA size is associated with a higher recurrence rate of AF treated with ablation [2]. However, not all AF patients with LA dilation will develop LASEC during TEE. Therefore, it was intriguing to find that LASEC may be a prognostic indicator in AF patients with LA dilation. The SEC intensities were classified into four grades (0–3) [4,12], and the prognostic implication of different grades was evaluated. It is believed that the outcomes of catheter ablation for AF patients with LASEC and LA dilation are unknown.

Patients with 45 mm ≤ LAD < 50 mm and LAV ≥ 120 mL were enrolled in this study. No significant differences in LAD, LAV, and CHA_2_DS_2_-VASc scores were observed between the LASEC and the no LASEC groups (*p* > 0.05).

After the 18-month follow-up period, the recurrence rate of AF/AT in the LASEC and no LASEC groups was 54.8% (40 patients) and 32% (16 patients), respectively. The difference in LASEC produced significant variations in the AF/AT recurrence-free survival between the two groups (log-rank test, *p* = 0.011). The presence of LASEC was associated with poor outcomes in the multivariate Cox regression analysis of AF/AT recurrence-free survival. These findings indicate that LASEC may be a predictor of AF recurrence after catheter ablation.

Akoum et al. [13] investigated the relationship between atrial fibrosis quantified using late gadolinium enhancement MRI (LGE-MRI) and TEE findings. The LA/LAA was classified as normal, presence of spontaneous echo contrast (SEC), or presence of thrombus. Atrial fibrosis was higher in patients with SEC (23.3 ± 13.7%) than in those without SEC (16.7 ± 10.8%; *p* = 0.01). The study found that atrial fibrosis is independently associated with spontaneous echo contrast. Moreover, Marrouche et al. [14,15] reported that the assessment of left atrial fibrosis with delayed enhancement MRI is associated with an increased risk of recurrent arrhythmia after catheter ablation for AF.

Although atrial fibrosis does not directly cause SEC, the tight association between them may render SEC an alternative prognostic indicator in health centers that are incapable of quantifying LA fibrosis. AF patients with LASEC and LA dilation may have a higher degree of atrial fibrosis, which predisposes these patients to recurrence after catheter ablation in the present study. Resultantly, AF patients with LASEC and LA dilation had poor prognoses following catheter ablation.

Meanwhile, during the follow-up, AF/AT was also found to recur in 30 (57.7%) and 15 (71.4%) cases in the mild and severe SEC groups, respectively. The prognosis declined with increasing SEC degree. This might be attributable to the fact that higher SEC grades are associated with worse atrial conditions and therefore poorer outcomes after catheter ablation.

The pathophysiology of thrombus formation can be primarily explained by alterations in Virchow’s triad: blood flow, hypercoagulability, and vascular endothelial injury [16,17]. SEC may fulfill the first two components of thrombogenesis [18]. No significant differences in D-dimer (0.27 ± 0.16 vs. 0.24 ± 0.18 mg/L) were observed between the LASEC and no LASEC groups (*p* > 0.05). However, the proportion of the flow velocity decrease in the LAA was higher in the LASEC group than in the no LASEC group (30.1% vs. 4%, *p* < 0.001).

During the follow-up, ischemic stroke occurred in two patients in the LASEC group due to discontinued anticoagulation. The CHA_2_DS_2_-VASc score of one patient was 1, due to female sex. Severe LASEC was detected on TEE before the ablation procedure for these two patients. Catheter ablation for AF patients with LASEC has a poor prognosis. This may be attributed to the fact that LASEC (especially severe LASEC) is an independent predictor of LAA thrombus formation and embolic events [19,20].

The present study has some limitations that must be considered for future analyses. First, it was a single-center, prospective study with a relatively small sample size. In the future, a more comprehensive study is needed with a larger study cohort. Second, silent embolism was not evaluated in this study.

## 5. Conclusions

LASEC could be a predictor of AF/AT recurrence after catheter ablation in AF patients with LA dilation. Higher degrees of LASEC are associated with poor prognosis.

## Figures and Tables

**Table 1:** Baseline demographic characteristics.

Characteristics	LASEC Group (*n* = 73)	No LASEC Group (*n* = 50)	*p* Value
Age (years)	62.6 ± 9.6	61.3 ± 8.9	0.319
Male sex (*n*, %)	47 (64.4%)	40 (80.0%)	0.062
PsAF (*n*, %)	72 (98.6%)	46 (92.0%)	0.173
History of AF (years)	3.2 ± 2.7	3.2 ± 2.9	0.779
Duration of AF (years) for PsAF	0.68 ± 0.45	0.73 ± 0.55	0.971
BMI (kg/m^2^)	25.4 ± 3.3	25.9 ± 2.9	0.359
DM (*n*, %)	14 (19.2%)	10 (20.0%)	0.910
Hypertension (*n*, %)	45 (61.6%)	37 (74.0%)	0.153
Stroke/TIA	8 (11.0%)	4 (8.0%)	0.815
Coronary artery disease (*n*, %)	7 (9.6%)	7 (14.0%)	0.449
Heart failure (*n*, %)	18 (24.7%)	12 (24.0%)	0.934
CHA_2_DS_2_-VASc score	2.3 ± 1.3	2.2 ± 1.5	0.308
Serum creatinine (μmol/L)	77.3 ± 13.9	80.3 ± 15.6	0.510
GFR (mL/min/1.73 m²)	84.9 ± 13.8	85.9 ± 16.8	0.707
D-dimers (mg/L)	0.27 ± 0.16	0.24 ± 0.18	0.059
Homocysteine (μmol/L)	12.7 ± 3.2	12.1 ± 2.7	0.554
Pro-BNP (pg/mL)	1088.6 ± 996.1	832.8 ± 887.9	0.068
Troponin I	0.02 ± 0.04	0.02 ± 0.01	0.233
LAD (mm)	47.1 ± 2.1	46.9 ± 2.0	0.598
LAV (cm^3^)	182.5 ± 43.9	172.8 ± 33.2	0.224
LVEDD (mm)	50.2 ± 4.6	50.4 ± 4.3	0.512
LVEF (%)	60.5 ± 8.3	61.7 ± 7.8	0.360

LASEC, left atrial spontaneous echo contrast; AF, atrial fibrillation; PsAF, persistent atrial fibrillation; BMI, body mass index; DM, diabetes mellitus; GFR, glomerular filtration rate; BNP, brain natriuretic peptide; LVEF, left ventricular ejection fraction; LAD, left atrial diameter; LAV, left atrial volume; LVEDD, left ventricular end-diastolic diameter; TIA, transient ischemic attack.

**Table 2:** Procedural details and medication use after the procedure.

	LASEC Group (*n* = 73)	No LASEC Group (*n* = 50)	*p* Value
Ablation energy (RFA/CBA)	53/20	33/17	0.433
Ablation strategy (PVI/PVI + LA roof line)	39/34	31/19	0.346
Total procedure duration (min)	125.1 ± 36.7	123.6 ± 33.0	0.903
Left atrial dwell time (min)	95.6 ± 36.1	96.2 ± 31.4	0.962
Total fluoroscopy time (min)	9.6 ± 4.2	8.8 ± 6.0	0.416
Radiation dose (μGym^2^)	2266.7 ± 1532.2	2348.4 ± 1822.6	0.655
Radiation dose (mGy)	210.2 ± 135.2	220.8 ± 170.3	0.518
Medication use after procedure			
Antiarrhythmic drug (*n*, %)	70 (95.9%)	47 (94.0%)	0.959
Beta-blocker (*n*, %)	21 (28.8%)	17 (34%)	0.537
NOAC (*n*, %)	62 (84.9%)	48 (96%)	0.239
ACEI/ARB (*n*, %)	43 (58.9%)	26 (52.0%)	0.449

LASEC, left atrial spontaneous echo contrast; CBA, cryoballoon ablation; RFA, radiofrequency ablation; PVI, pulmonary vein isolation; LA, left atrial; NOAC, nonvitamin K antagonist oral anticoagulant; ACEI/ARB, Angiotensin-converting enzyme inhibitors and angiotensin receptor blockers.

**Table 3:** Contingency table with incidence of AF/AT recurrence at 18 months.

	No AF/AT Recurrence	With AF/AT Recurrence
LASEC group	33 (45.2%)	40 (54.8%)
No LASEC group	34 (68.0%)	16 (32.0%)

Incidence of AF/AT recurrence at 18 months; Pearson chi-square, *p* = 0.013. LASEC, left atrial spontaneous echo contrast; AF/AT, atrial fibrillation/atrial tachycardia.

**Table 4:** Univariate and multivariate Cox regression analysis of freedom from AF/AT recurrence-free survival (time to AF/AT recurrence is the dependent variable).

Total Population (*n* = 123)	Univariate Analysis			Multivariate Analysis		
	*p*	HR	95% CI	*p*	HR	95% CI
Age (≥65 vs. <65 years)	0.611	0.872	0.516–1.475			
Sex (male vs. female)	0.621	1.161	0.642–2.097			
BMI (abnormal vs. normal)	0.217	0.719	0.425–1.215			
CHA_2_DS_2_-VASc score (>2 vs. ≤2)	0.932	1.023	0.603–1.737			
LAD (≥47 vs. <47 mm)	0.122	0.658	0.387–1.118			
HF (HF vs. normal)	0.011	0.379	0.179–0.802	0.008	0.359	0.168–0.768
LAV (≥170 vs. <170 cm^3^)	0.025	1.859	1.082–3.196	0.040	1.787	1.028–3.108
History of AF (≥2 vs. <2 years)	0.003	3.005	1.471–6.137	0.018	2.415	1.166–5.001
LASEC (LASEC vs. no LASEC)	0.015	2.058	1.151–3.679	0.009	0.455	0.253–0.819
Ablation energy (CBA vs. RFA)	0.411	1.261	0.725–2.193			
Ablation strategy (PVI vs. PVI + LA roof line)	0.905	1.035	0.590–1.813			

LASEC, left atrial spontaneous echo contrast; CBA, cryoballoon ablation; RFA, radiofrequency ablation; PVI, pulmonary vein isolation; AF, atrial fibrillation; HF, heart failure; BMI, body mass index; LAD, left atrial diameter; LAV, left atrial volume; AF/AT, atrial fibrillation/atrial tachycardia.

## Data Availability

The data presented in this study are available on reasonable request.
